# Inflammation to Pulmonary Diseases

**DOI:** 10.1155/2016/7401245

**Published:** 2016-05-16

**Authors:** Kang-Yun Lee, Kazuhiro Ito, Kittipong Maneechotesuwan

**Affiliations:** ^1^Division of Pulmonary Medicine, Shuang Ho Hospital and Division of Pulmonary Medicine, School of Medicine, College of Medicine, Taipei Medical University, 250 Wuxing street, Taipei 11031, Taiwan; ^2^Airway Disease, National Heart and Lung Institute, Imperial College London, Dovehouse street, London SW7 6LY, UK; ^3^Faculty of Medicine Siriraj Hospital, Mahidol University, Wanglang Street, Bangkok 10700, Thailand

Inflammation, which is induced and perpetuated by microorganism pathogens or from the damage or death of host cells, is an essential component of various common respiratory diseases, such as chronic obstructive pulmonary disease (COPD), asthma, bronchiectasis, and acute respiratory distress syndrome (ARDS), as well as of less common ones, for example, sarcoidosis. Although different diseases express distinct inflammatory responses, there are some shared common features. For example, the Th2-related eosinophilic inflammation, which is typically present in asthma, is also characteristic of eosinophilic phenotype of COPD. On the other hand, the innate neutrophilic inflammation shared by a number of diseases, including COPD and ARDS, could be demonstrated in some patients with severe asthma. In this special issue, common inflammation was dissected in diverse ways by the authors to better understand the related respiratory diseases, from the clinical to the underlying mechanisms, pointing to future therapeutic prospect.

Innate immunity plays a primary role in host defense against microorganism pathogens and in response to the damage or death of host cells; however, when dysregulated, instead, it changes to an essential component of various respiratory diseases. In this issue, neutrophilic inflammation was demonstrated to be involved in two clinical conditions poorly understood. F. Tang et al. investigated the neutrophilic inflammation in patients with suboptimally controlled asthma and observed a defective innate immunity, which might be linked to pathogen-induced exacerbations. F. L. Dente et al. suggested sputum neutrophilic inflammation as a good biomarker for disease severity of stable noncystic fibrosis bronchiectasis by careful investigation of correlation with clinical parameters in those subjects. K. Baines et al. unearth the role of the innate immune antimicrobial peptide *β*-defensin-1 in COPD and severe asthma, which is involved in persistent inflammation in those conditions and they also suggested it as a potential biomarker in sputum and a therapeutic target. Using similar approach, A. K. Barton et al. correlated BAL MMPs/TIMPs with clinical and cytological findings in different equine chronic pneumopathies and suggested that their ratios can be a good biomarker for disease severity and used for identifying subclinical cases.

A number of the articles addressed the regulatory mechanisms or novel therapies for ARDS, a disease with overwhelming nonspecific inflammation evoked by variable etiology. L. Ma et al. tested the protective roles of 3,5,4′-tri-O-acetylresveratrol (AC-Rsv), a prodrug of resveratrol, in LPS-induced ARDS, focusing on the modulation of SIRT1 and the mitogen-activated protein kinase (MAPK) pathway. In a review article, Z. Xu et al. on the other hand gave attention to the dark side of histones, the extracellular histones, acting as new members of damage-associated molecular pattern (DAMP) molecules. C. F. Gonçalves-de-Albuquerque et al. extensively reviewed the oleic acid-induced lung injury and ARDS. In addition to discussing the featured pathophysiological changes and the underlying pathogenesis, they also shed light on a link between lipid metabolism and inflammatory diseases. Finally, W.-C. Chou et al. reported a therapeutic role of caffeine in mitigating acute lung injury induced by ischemia-reperfusion of lower limbs.

To combat the pathogens evading the innate response, the immune system mounts adaptive immunity. The immune response to tuberculosis is such a prototype, for example, Th1 immune response. However, in the absence of known etiology, granulomatous inflammation characterized with infiltration of activated Th1/Th17 lymphocytes as well as macrophages features the pathological changes of pulmonary sarcoidosis. By using correlation network analysis into BAL cells in sarcoidosis, T. Dyskova et al. addressed the contribution of both microRNAs and the Th1-transcription factor T-bet to the regulation of cytokine/chemokine-receptor network, which drives the inflammatory granulomatous disease.

Thus, inflammatory pulmonary diseases, as shown in this special issue, usually recapture the inflammation evoked by the immune system to the invading pathogens or other environmental stress. Thus, one mechanism might underlie variable diseases, directing to a common therapy.

*Kang-Yun Lee* *Kang-Yun Lee* *Kazuhiro Ito* *Kazuhiro Ito* *Kittipong Maneechotesuwan* *Kittipong Maneechotesuwan*

